# Surgical Treatment of a Patient with a Solitary Thyroid Metastasis from Primary Sigmoid Adenocarcinoma: A Case Report and Literature Review

**DOI:** 10.1155/2019/3208469

**Published:** 2019-05-26

**Authors:** Kohei Yamahara, Toshiaki Moriki, Yuki Katsura, Kana Lee, Satoshi Ikegami

**Affiliations:** ^1^Department of Otolaryngology, Head and Neck Surgery, Shizuoka City Shizuoka Hospital, Shizuoka 420-8630, Japan; ^2^Department of Pathology, Shizuoka City Shizuoka Hospital, Shizuoka 420-8630, Japan; ^3^Department of Otolaryngology, Shin-Suma General Hospital, Kobe, Hyogo 654-0048, Japan

## Abstract

Malignant metastases to the thyroid are rare and even rarer from colorectal cancer (CRC). Most cases of CRC metastasis to the thyroid involve metastases to other organs as well, particularly the liver and/or lung. There are only three reports of CRC metastasizing to the thyroid without involvement of another site. Patients with solitary thyroid metastasis from CRC have a poor prognosis after surgery, whereas resection is beneficial in their counterparts with a solitary liver or lung metastasis. This difference could be the result of delayed diagnosis of thyroid metastasis in patients with CRC, given that postoperative follow-up examination of the thyroid is not routinely performed. Here we describe a patient who was found to have a solitary metastasis of sigmoid cancer to the thyroid on postoperative imaging and has had prolonged disease-free survival after thyroidectomy. Our experience suggests that a low threshold of suspicion is crucial for timely diagnosis of thyroid metastasis from CRC and that resection can improve disease-free survival.

## 1. Introduction

Thyroid metastases are rare, with renal cell carcinoma being the most frequent cause in clinical series [1]. Thyroid metastases from CRC are even rarer; one report identified only 6 cases among 5862 patients with CRC (0.1%) between 1993 and 2004 [2]. Almost all cases with metastasis of CRC to the thyroid have involvement of other organs, in particular the liver and/or lung. There are only three reports of CRC metastasis to the thyroid without involvement of another site [3–5]. Surgical resection is generally considered in patients with a solitary liver and/or lung metastasis from CRC [6]. However, patients with a solitary thyroid metastasis from CRC reportedly have a poor prognosis after thyroidectomy, so surgical treatment is controversial [7]. Routine follow-up examinations of the thyroid for metastases from CRC are not usually performed after colon surgery. Therefore, delayed diagnosis could account for the poor prognosis in patients who develop a solitary thyroid metastasis after surgery.

In this report, we describe a patient in whom a solitary metastasis of sigmoid cancer was found in the thyroid gland in the course of 6-month postoperative radiologic follow-up. The patient remains disease-free 24 months after thyroid lobectomy.

## 2. Case Report

**Figure 1:**
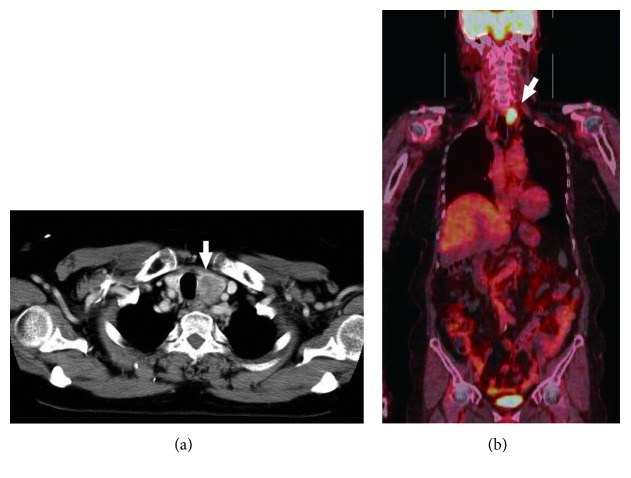
Enhanced CT and PET-CT before surgery. CT demonstrates spread of the mass to the tracheoesophageal groove, suggesting invasion of the left recurrent laryngeal nerve (a). PET-CT shows focal uptake in the left thyroid lobe, with no evidence of distant metastasis (b). The mass is indicated by arrows. CT, computed tomography; PET, positron emission tomography.

**Figure 2:**
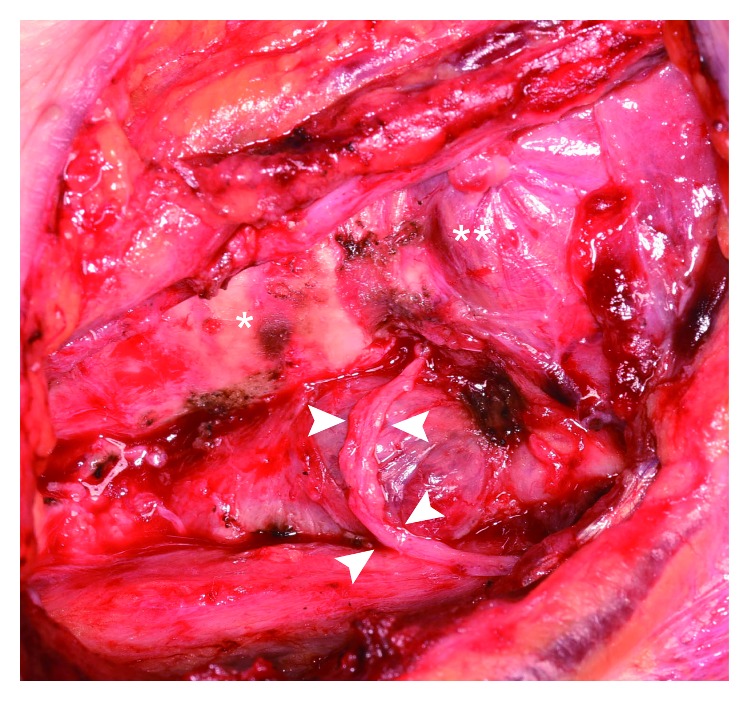
The operative field after left hemithyroidectomy with resection of the left RLN. The left RLN was cut around its entry to the larynx. We then performed an ansa cervicalis-to-RLN anastomosis to reinnervate the left vocal fold. The arrows indicate the anastomosis. The asterisk indicates the trachea. The double asterisk indicates the cricothyroid muscle. RLN, recurrent laryngeal nerve.

**Figure 3:**
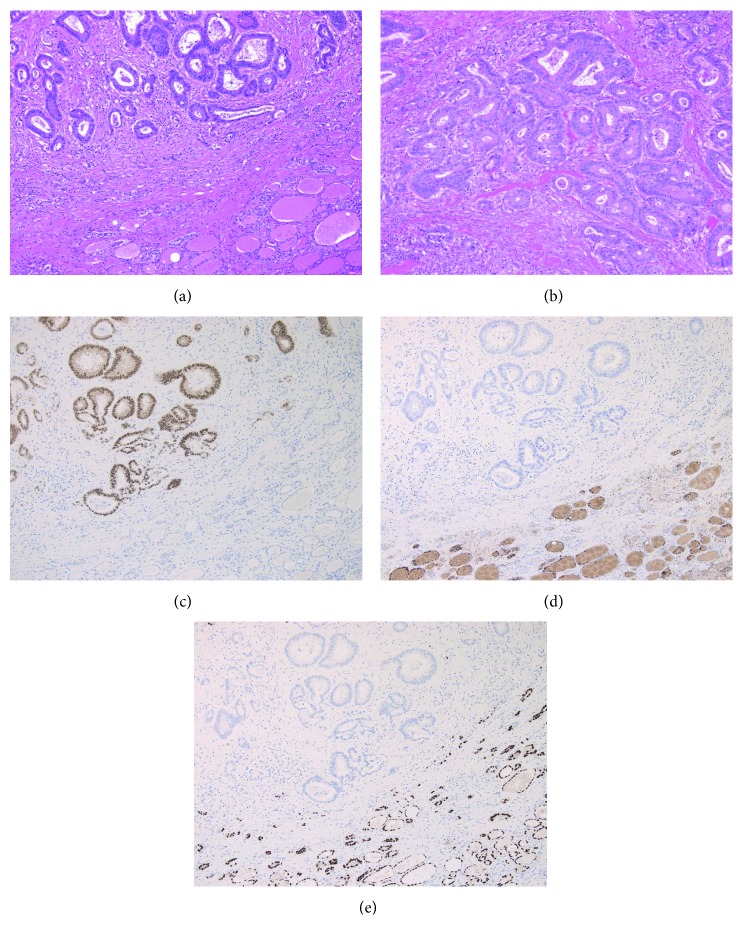
Pathologic examination of the excised mass. Hematoxylin and eosin staining of excised mass showed a normal thyroid goiters and adenocarcinoma (a) similar to that in the sigmoid cancer specimen (b). Immunohistochemistry of CDX2 for excised mass was positive for adenocarcinoma and negative for thyroid goiters (c), while immunohistochemistry of both thyroglobulin (d) and TTF-1 (e) was negative for adenocarcinoma and positive for thyroid goiters.

The patient was a 74-year-old woman who had undergone colectomy for adenocarcinoma of the sigmoid colon at the age of 72 years. Before the colectomy, she had been found to have a tumor measuring approximately 25 mm in the left lobe of the thyroid that was diagnosed as an adenomatous goiter by fine-needle aspiration. Two years after her surgery, a 6-month follow-up computed tomography (CT) scan revealed enlargement of the thyroid tumor, but she remained asymptomatic. Blood tests revealed a small increase in CA 19-9 (from 3.5 ng/ml 6 months earlier to 8.9 ng/ml) and in carcinoembryonic antigen (CEA) (from 1.7 ng/ml to 4.6 ng/ml). Her thyroid function tests were normal. Physical examination and laryngoscopy revealed a firm elastic nodule in the thyroid gland and left vocal fold paralyzed in the midline position. The maximum phonation time (MPT) was 10 seconds. There was no cervical lymphadenopathy. Ultrasonographic examination of the neck revealed a solid tumor in the left thyroid lobe with a diameter of 35 × 25 × 20 mm. CT showed spread of this mass to the tracheoesophageal groove, suggesting invasion of the left recurrent laryngeal nerve (RLN; Figure 1(a)). Fine-needle aspiration cytology of the thyroid tumor showed a few clusters of elongated tumor cells with hyperchromatic dark nuclei on a background of benign hepatocytes, and the mass was reported as metastatic adenocarcinoma. Positron emission tomography-CT showed focal uptake in the left thyroid lobe with no evidence of distant metastasis (Figure 1(b)). Therefore, the diagnosis was metastasis of adenocarcinoma to the left thyroid gland. We then performed a hemithyroidectomy with resection of the left RLN and immediate reconstruction using the ansa cervicalis nerve (Figure 2). The tumor was observed to be adherent to the adjacent structures, i.e., the trachea and external muscle of the esophagus as well as the left RLN. The surgical margin was confirmed to be adequate, and the decision was made not to resect the right thyroid lobe. There were no postoperative complications, and the patient was discharged home 5 days after surgery. Hematoxylin and eosin staining of the tumor showed a normal thyroid goiter and adenocarcinoma similar to that in the sigmoid cancer specimen (Figures 3(a) and 3(b)). The adenocarcinoma was occupying almost the entire left lobe of the thyroid. Immunohistochemistry showed that the adenocarcinoma was positive for CDX2 and negative for thyroglobulin or TTF-1 (thyroid transcription factor 1) (Figures 3(c)–3(e)), which confirmed a colorectal origin. The diagnosis was solitary metastasis of sigmoid cancer to the thyroid gland. CEA and CA19-9 levels returned to normal within 2 months postoperatively. Adjuvant chemotherapy with uracil-tegafur/leucovorin was initiated, and 24 months after surgery the patient remains alive without recurrence. She got a breathy voice with MPT of 6 seconds immediately after surgery, but her vocal fold tone has recovered to MPT of 10 seconds.

## 3. Discussion

Thyroid metastasis from a nonthyroid site is uncommon and comprises only 1.4%–3% of all thyroid neoplasms [8]. The lung has been reported to be the most common site of metastasis of a primary tumor to the thyroid in autopsy series, whereas renal cell carcinomas, followed by breast and gastrointestinal neoplasms, have been found to be the most common primary tumors causing thyroid metastases in clinical series [9]. According to Willis, the high oxygen and iodine environment in the thyroid may impair the ability of metastatic cells to settle and develop at this site [1]. Furthermore, rapid blood flow could make it difficult for tumor cells to adhere and implant at this site [1]. Willis also reported that adenomatous and other retrograde structural changes in the thyroid tissues predispose to metastasis, as in our case, because altered vascular conditions in adenomatous or abnormal areas may favor trapping of blood-borne emboli [1]. Moreover, Smith et al. investigated 19 patients with carcinoma metastatic to the thyroid at their institution and reported that 11 had a preexisting abnormality, i.e., adenomatous, multinodular, or lymphocytic goiter, in the thyroid before detection of metastasis [10].

Thyroid metastasis of CRC is rare, but an increasing number of cases are being reported. Keranmu et al. identified 21 patients with adequately detailed information available [11]. The majority of reported cases of CRC metastasis to the thyroid have had involvement of other major organs. CRC is considered to metastasize to the thyroid gland mainly via the hematogenous pathway, i.e., the portal vein, vena cava, and pulmonary vein [8]. However, a search of the relevant PubMed literature revealed only three cases of metastasis of colon cancer to the thyroid without involvement of another site (Table 1). The mechanism proposed for this solitary metastasis is that the vertebral venous system allows the primary tumor to bypass the portal vein, pulmonary vein, and vena cava and to transfer directly to the thyroid or another site in the body without entering the thoracic or abdominal cavity [12].

Generally, thyroid metastasis of CRC is associated with high mortality, although the prognosis may be determined by multiple factors, including the grade of malignancy of the primary lesion and whether the metastases are diagnosed in a timely manner. Keranmu et al. reported that the median survival time after thyroid metastasis during 3 years of follow-up was 12 months [11]. A solitary liver or lung metastasis of CRC is usually resected because it is likely to extend survival [6]. However, as shown in Table 1, the survival times in the three patients in whom solitary thyroid metastasis of CRC has been documented were only 2, 9, and 10 months after surgery. Moreover, disease-free survival was only 5 and 7 months in two cases (tracheostomy was only performed on one patient). Keranmu et al. concluded that there was no significant difference in overall survival between patients treated nonsurgically and those who undergo thyroidectomy [11]. Therefore, surgical treatment of such patients is still controversial [7].

However, our patient has so far had a disease-free interval of 24 months since thyroidectomy, which is markedly longer than that in previous reports and may reflect early detection of her thyroid metastasis by follow-up imaging. Most metastases to the thyroid are asymptomatic, so accurate imaging examinations are essential for early detection. In the case reported by Choufani et al., the patient's CA19-9 and CEA levels were increasing in 1991, but they could not find the reason for the increase in these tumor markers for 3 years, possibly because of lack of use of PET-CT and lack of suspicion of thyroid metastasis of CRC [4]. Thyroid metastasis was found in a case reported by Wychulis et al. in 1940 and in a case reported by Sklaroff et al. in 1952. These older reports suggest that early detection was missed because of unavailability of accurate imaging techniques at that time [3, 5]. Therefore, the metastatic tumors might have been too advanced to allow complete resection in these three cases. Current European and US guidelines for colon cancer recommend postoperative CT only once a year [13, 14]. However, in Japan, clinicians usually obtain CT scans of the chest and abdomen, which cover the lower part of the neck, every 6 months postoperatively. Therefore, we were able to detect slight enlargement of the thyroid tumor and achieve a complete tumor resection in our patient. This case suggests that clinicians should have a low threshold of suspicion for thyroid metastasis of CRC and that frequent follow-up imaging is crucial to making a timely diagnosis. Moreover, our experience suggests that surgical resection should be performed if an isolated metastatic CRC to the thyroid is found because it could prolong disease-free survival and, as for a solitary liver or lung metastasis of CRC, may even be curative.

There is no consensus concerning the extent of thyroidectomy for thyroid metastasis from a nonthyroid site. Total thyroidectomy seems to be a good treatment option to avoid leaving behind residual thyroid tissue that may harbor neoplastic foci; however, Calzolari et al. demonstrated that the difference in survival between patients submitted to total thyroidectomy and those who underwent hemithyroidectomy was not statistically significant, indicating that the extension of surgical resection does not have a significant impact on survival [8]. Moreover, adverse events of surgery, such as hypocalcemia or recurrent laryngeal nerve paralysis, were more frequent and severe in association with total thyroidectomy than hemithyroidectomy [15]. In our patient, we confirmed that there was no apparent mass in the right thyroid lobe by preoperative examinations: ultrasonography, CT, or PET-CT. In addition, intraoperative histologic assessment of surgical margin was negative; thus, we performed hemithyroidectomy, instead of total thyroidectomy. Our case suggests that extent of surgical resection of the thyroid should be determined by extent of the tumor's spread for the treatment of thyroid metastasis from a nonthyroid site.

In our patient, the left RLN was invaded by the metastatic tumor and cut around its entry to the larynx. A denervated vocal fold will become atrophied over time, leading to hoarseness even if it is paralyzed in the midline. Therefore, we performed an ansa cervicalis-to-RLN anastomosis to reinnervate the left vocal fold. Several methods can be used to reestablish the neural pathway to the vocal fold, including a nerve-muscle pedicle, a muscle-nerve-muscle pedicle, and a donor nerve-RLN anastomosis. The most researched procedure is the ansa cervicalis-RLN anastomosis, which has the advantages of providing vocal fold tone, bulk, and tension and being technically simple to perform. The ansa cervicalis is an ideal choice for a donor nerve because of its readily accessible location near the laryngeal complex and its relatively constant resting tone. Lorenz et al. reviewed the 38 patients who underwent ansa cervicalis-RLN anastomosis at their institution and reported that 37 demonstrated clinical evidence of reinnervation 6 months after surgery [16]. An ansa cervicalis-RLN anastomosis takes several months to achieve reinnervation and may not be indicated in patients with metastasis of CRC to the thyroid because of their expected poor prognosis. However, our patient has so far survived for 24 months after surgery, and her vocal fold tone seems to have recovered in this time, indicating that it was appropriate to perform an ansa cervicalis-RLN anastomosis in this case.

## 4. Conclusion

We have presented a rare case of solitary metastasis of sigmoid cancer to the thyroid gland. Unlike in the three previous reports of solitary thyroid metastasis of CRC, our patient is doing well 24 months following surgery, suggesting that surgical resection with adjuvant therapy may be appropriate for better outcome when a solitary thyroid metastasis of CRC is early detected.

## Figures and Tables

**Table 1:** Three cases of CRC metastasis to the thyroid without involvement of another site.

Authors/years^*∗*^	Age (year)/sex	Primary site	Disease-free survival period after thyroidectomy (months)	Surgical procedure	Prognosis (months)^*∗∗*^
Wychulis [3]/1940	37/F	R	7	Subtotal thyroidectomy	Dead (10)
Sklaroof [5]/1952	73/F	R	—	Tracheostomy	Dead (2)
Choufani [4]/1991	75/F	SC	5	Total thyroidectomy	Dead (9)

R and SC indicate rectum and sigmoid colon, respectively. ^*∗*^The time when thyroid metastasis occurred. ^*∗∗*^Survival period after thyroid metastasis.

## References

[1] Willis R. A. (1931). Metastatic tumours in the thyreoid gland. *American Journal of Pathology*.

[2] Lièvre A., Leboulleux S., Boige V. (2006). Thyroid metastases from colorectal cancer: the Institut Gustave Roussy experience. *European Journal of Cancer*.

[3] Wychulis A. R., Beahrs O. H., Woolner L. B. (1964). Metastasis of carcinoma to the thyroid gland. *Annals of Surgery*.

[4] Choufani E. B., Lazar H. L., Hartshorn K. L. (2001). Two unusual sites of colon cancer metastases and a rare thyroid lymphoma. Case 2. Chemotherapy-responsive right artial metastasis from colon carcinoma. *Journal of Clinical Oncology*.

[5] Sklaroff D. M. (1954). Metastatic carcinoma of rectum to the thyroid gland: correlation with radioactive iodine studies. *AMA. Archives of Surgery*.

[6] Goonerante D., Gray C., Lim M. (2013). Survival outcome in New Zealand after resection of colorectal cancer lung metastases. *ANZ Journal of Surgery*.

[7] Froylich D., Shiloni E., Hazzan D. (2013). Metachronous colon metastasis to the thyroid: a case report and literature review. *Case Reports in Surgery*.

[8] Calzolari F., Sartori P. V., Talarico C. (2008). Surgical treatment of intrathyroid metastases: preliminary results of a multicentric study. *Anticancer Research*.

[9] Nixon I. J., Coca-Pelaz A., Kaleva A. I. (2017). Metastasis to the thyroid gland: a critical review. *Annals of Surgical Oncology*.

[10] Smith S. A., Gharib H., Goellner J. R. (1987). Fine-needle aspiration. Usefulness for diagnosis and management of metastatic carcinoma to the thyroid. *Archives of Internal Medicine*.

[11] Keranmu A., Zheng H., Wu Y. (2017). Comparative study of single-center patients with thyroid metastases from colorectal cancer and previously reported cases in the literature. *World Journal of Surgical Oncology*.

[12] Batson O. V. (1940). The function of the vertebral veins and their role in the spread of metastases. *Annals of Surgery*.

[13] Benson A. B., Venook A. P., Cederquist L. (2017). Colon cancer, version 1.2017, NCCN clinical practice guidelines in oncology. *Journal of the National Comprehensive Cancer Network*.

[14] Desch C. E., Benson A. B., Somerfield M. R. (2005). Colorectal cancer surveillance: 2005 update of an American Society of Clinical Oncology practice guideline. *Journal of Clinical Oncology*.

[15] Kuba S., Yamanouchi K., Hayashida N. (2017). Total thyroidectomy versus thyroid lobectomy for papillary thyroid cancer: comparative analysis after propensity score matching: a multicenter study. *International Journal of Surgery*.

[16] Lorenz R. R., Esclamado R. M., Teker A. M. (2008). Ansa cervicalis-to-recurrent laryngeal nerve anastomosis for unilateral vocal fold paralysis: experience of a single institution. *Annals of Otology, Rhinology & Laryngology*.

